# Lurasidone uses and dosages in Spain: RETROLUR, a real-world retrospective analysis using artificial intelligence

**DOI:** 10.3389/fpsyt.2024.1506142

**Published:** 2025-02-05

**Authors:** Fernando Mora, Carlos Gómez Sánchez-Lafuente, Mariano De Iceta, Carolina Roset, Antonio Cárdenas, Daniel Pérez, Elena Álvarez-Barón, Irene Gabarda-Inat, Savana Research Group

**Affiliations:** ^1^ Department of Psychiatry and Mental Health, Hospital Universitario Infanta Leonor, Madrid, Spain; ^2^ Department of Legal Medicine, Psychiatry and Pathology, Complutense University, Madrid, Spain; ^3^ Department of Mental Health, Hospital Regional Universitario de Málaga, Andalucía, Spain; ^4^ Hospital Universitario Infanta Sofía, S.S.Reyes, Madrid, Spain; ^5^ Universidad Europea de Madrid, Faculty of Medicine, Health and Sports, Digital Department of Biomedical and Health Sciences, Madrid, Spain; ^6^ Fundación para la Investigación e Innovación Biomédica, Hospital Universitario Infanta Sofía, H.U. del Henares y H.U. del Tajo, Madrid, Spain; ^7^ Department of Psychiatry, Hospital Universitario Son Espases, Islas Baleares, Spain; ^8^ Medical Department, Angelini Pharma España SLU, Barcelona, Spain; ^9^ Global Medical Department, Angelini Pharma S.p.A., Rome, Italy

**Keywords:** lurasidone, schizophrenia, bipolar depression, Spain, electronic health records

## Abstract

**Introduction:**

Lurasidone is used for schizophrenia and bipolar depression in many countries, yet there is a lack of existing literature about its use, efficacy, and safety in real life. We aimed to characterize lurasidone-treated patients by analyzing unstructured information in electronic health records (EHRs).

**Methods:**

This was a multicenter, retrospective, observational, and descriptive study that used data extracted from EHRs of patients initiating treatment with lurasidone in four Spanish hospitals from September 2019 to March 2022. Stratification included primary diagnosis, antipsychotic therapy, and lurasidone dose. Natural language processing and machine learning were used to extract and analyze unstructured clinical data using SNOMED CT terminology. Sociodemographic, clinical, and treatment characteristics, as well as symptoms and potential adverse events as efficacy and safety outcomes, were evaluated at inclusion and during follow-up.

**Results:**

Among 2,374,218 patients attending the participating hospitals during the study period with 66,523,391 EHRs, 272 initiated lurasidone and were included. Median (Q1; Q3) age was 46 (37; 56) years, and 60.3% were female. Common comorbidities were hypertension (46.7%), dyslipidemia (44.5%), and diabetes (30.5%), and 87.1% had received a median of three antipsychotics before lurasidone, being olanzapine (52.9%) and quetiapine (45.2%) the most frequently used. During follow-up, 16.9% of the patients discontinued lurasidone, and few patients (<1.2%) reached high doses (111 and 148 mg/day). Lurasidone demonstrated effectiveness in reducing positive and negative symptoms, anxiety, depression, and suicidal ideation, with a marked reduction in most of the adverse events compared to the pre-lurasidone period.

**Discussion:**

Lurasidone reduced positive and negative symptoms frequencies with a favorable safety profile, while low discontinuation rates suggest efficacy-tolerability balance, patient satisfaction, and acceptability. Our data reflect that in Spain lurasidone is used at low doses, limiting its beneficial effects according to clinical trials results.

## Introduction

Antipsychotics are often a first-line treatment for psychiatric disorders such as schizophrenia and bipolar depression (1). The use of second-generation, or atypical, antipsychotics has gained popularity, and their efficacy and safety appear to rival or surpass first-generation antipsychotics (2). Lurasidone is a second-generation oral antipsychotic that was approved by the U.S. Food and Drug Administration (FDA) in 2010 and 2013 for schizophrenia and acute bipolar depression in adults, respectively, either as monotherapy or in combination (3). It was later approved by the European Medicines Agency (EMA) for schizophrenia treatment in adults in 2014 and for adolescents aged 13 to 17 in 2020 (4). Lurasidone has demonstrated safety and efficacy in short- and long-term studies for the treatment of schizophrenia (37-148 mg/day) and bipolar depression (18.5-111 mg/day), with minimal metabolic effects and low weight gain risk (5–7). Systematic reviews have extensively analyzed its efficacy, tolerability, safety, and place in therapy (8, 9). A few meta-analyses suggest that lurasidone has a similar efficacy profile to other antipsychotics, and it offers several advantages, including ease of practical use, early efficacy, and tolerability, particularly cardiometabolic (10, 11). Additionally, lurasidone has been shown to be more effective in treatment-naive patients (12, 13). Despite these studies, there is a lack of existing literature on the real-world use of lurasidone (14, 15).

Describing the patients receiving psychotropic therapies has been crucial as a first step toward understanding this patient population for future comparative studies (16–21), whether focusing on efficacy (22–24), safety (25, 26), or the relevance of monotherapy versus polytherapy (27). While clinical trials are critical for evidence generation, they have limitations in capturing real-world complexities due to controlled settings and participant numbers. Real-world data (RWD) studies, on the other hand, offer a more comprehensive and practical view of the outcomes of medical treatments. Although recent studies have reported RWD on the schizophrenia epidemiology in the Spanish population, they rely on structured data from the Minimum Basic Data Set (MBDS), or coding systems such as the International Classification of Diseases (ICD) or the Anatomical Therapeutic Chemical Classification System (ATC) (28). In this regard, unstructured free text from electronic health records (EHRs) provides richer clinical insights (29, 30). However, since lurasidone’s approval, its use in routine clinical practice has not been extensively documented, and there is a growing interest in how lurasidone might be combined with other antipsychotics in real practice (31). Therefore, it is essential to describe those patients taking lurasidone and the characteristics of its use in our country to enhance our understanding of this clinical population.

This study aimed to portray the clinical characteristics and medical management of patients treated- with lurasidone in Spain either as a stand-alone treatment or in conjunction with other antipsychotics, based on RWD and by analyzing readily available unstructured information recorded by healthcare professionals in patients’ EHRs. To this end, the study relied on using natural language processing (NLP) and machine learning (ML) techniques to extract, organize, and analyze this clinical information to better understand its use, efficacy, and safety in Spanish patients as a step to support clinicians in their decision making when prescribing antipsychotics.

## Methods

### Study design and data source

**Figure 1:**
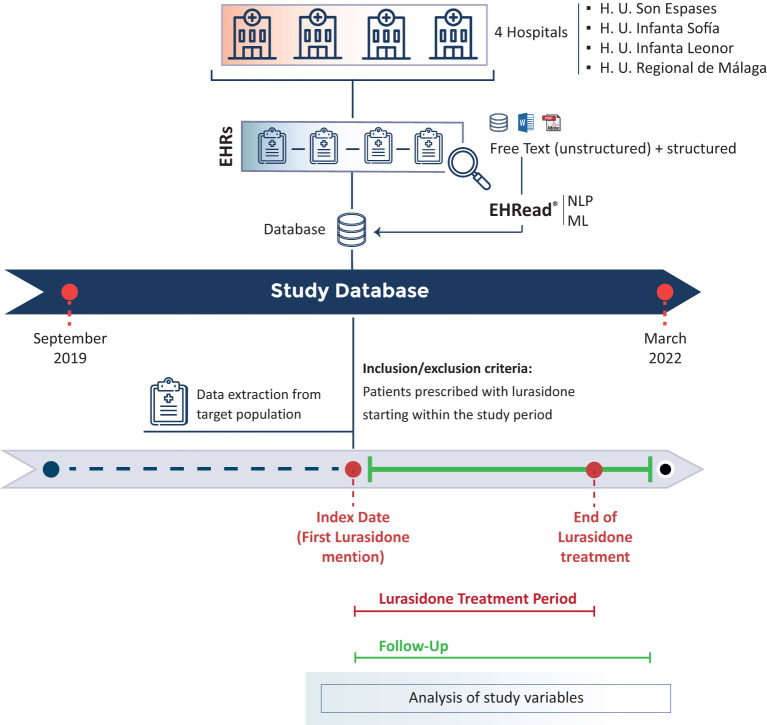
Study design. EHRead^®^ technology by Savana is a system based on Natural Language Processing (NLP) that applies machine learning (ML) to extract, analyze, and interpret the free-text information written in millions of de-identified EHRs. The unstructured and structured free-text information from EHRs from multiple participating sites is organized in study databases. Specific inclusion and exclusion criteria are specified to define the target population. The variables extracted from the database at Index date (baseline), during the follow-up period, and throughout the Lurasidone Treatment Period are organized and analyzed to address multiple clinical questions.

This was a multicenter, observational, and descriptive study in which RWD captured in the EHRs of patients prospectively attended and treated with lurasidone in four Spanish hospitals from September 2019 to March 2022, were retrospectively collected (**Figure 1**). A cross-sectional analysis of all patients and variables was performed at the time of inclusion with a variable-dependent look-back window as described in the table footnotes. These time windows were intended to establish a reasonable time interval at which the occurrence of variables could be related to a specific time point. Analyses were also performed during the follow-up period (i.e., the time from inclusion to the last EHR available within the study period or from inclusion to the end of treatment with lurasidone). Sample size calculation as well as more details regarding data source, acquisition, integration and quality assessment are shown in the Supplementary Materials.

### Study population

The study population included all patients attending the participating hospitals who initiated lurasidone treatment during the study period according to unstructured (and structured, if available) information in the EHRs. To avoid the inclusion of false positive or non-informative patients, to be considered part of the study population, two independent mentions of lurasidone had to be identified in different EHRs, and at least one of them had to be from the psychiatry department.

There were no exclusion criteria. Included patients were further stratified into different subgroups concerning primary diagnosis (i.e., schizophrenia, schizoaffective disorder, bipolar disorder, psychotic episode, depression, or not specified), maximum dose of lurasidone (18.5 mg, 37 mg, 74 mg, 111 mg, 148 mg or not specified) and type of antipsychotic therapy (lurasidone monotherapy or polytherapy).

### Data extraction

Unstructured clinical data were extracted from all available departments (including inpatient, outpatient, and emergency departments) in all included patients at each participating site and analyzed using the EHRead^®^ technology (Medsavana, Madrid, Spain) according to previously described methods (32). Briefly, the free text information from EHRs was extracted and organized using the SNOMED CT terminology. This data-driven methodology relies on NLP and ML to generate a synthetic database containing any detection of medical concepts and associated metadata (33, 34). We evaluated the performance of EHRead^®^ as previously described (35). Details regarding EHRead^®^ data extraction, model development and specific metrics obtained after the external validation are in Supplementary Materials and **Supplementary Table 1**.

### Study variables

Variable categories included sociodemographic characteristics, toxic habits, comorbidities, clinical characteristics, diagnosis, lurasidone characteristics (timing, dosage, concomitant medication, relapses, etc), other antipsychotics and effectiveness (symptoms), and safety outcomes (potential treatmen-related adverse events). Because this study relied on RWD, the potential number of variables included in the analyses was limited by the free text information contained in the EHRs. See Supplementary Materials for further details.

### Statistical analysis

Frequency tables were used for categorical variables, while continuous variables were described using summary tables which may include the mean, standard deviation (SD), median, and the lower and upper quartiles (Q1; Q3). The number of non-evaluable outcomes and missing data were also reported and not counted in the percentages. The Kaplan-Meier (KM) approach was used for time-to-event analyses. Missing data is detailed in the Supplementary Materials and was handled according to the nature of the data collection process and based on the type of variable (boolean, categorical or numerical) assuming that physicians reflect clinically relevant information in EHRs. Then, missing data imputation was applied to boolean variables, treating the absence of a term for a comorbidity/symptom/adverse event as if the patient did not have it. Data were analyzed and presented using “R” software (version 4.0.2).

### Ethical and regulatory considerations

This study was classified as a “non-interventional post-authorization study” by the Spanish Agency for Medicines and Health Products (AEMPS) and was reviewed and approved by the Institutional Review Board (IRB) of each participating hospital. It followed a predefined protocol and statistical analysis plan that was designed to ensure the scientific integrity of the research. All methods and analysis followed legal and regulatory requirements and generally accepted research practices as described in the latest edition of the Declaration of Helsinki, Good Pharmacoepidemiology Practices, and applicable local regulations. Informed consent was waived because data were retrospectively analyzed from patient EHRs, anonymized, and aggregated in an irreversibly dissociated manner. Data registry in EHRs was performed as part of routine clinical practice. Data was further included in the study for its secondary use in research and its assessment was blinded.

## Results

### Study population and baseline characteristics

**Figure 2:**
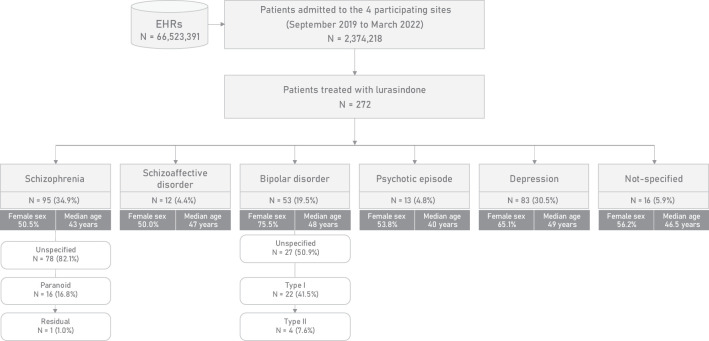
Flowchart of the study population and stratification by primary diagnosis.

A total of 66,523,391 EHRs containing clinical data on 2,374,218 patients who attended the participating hospitals during the study period were processed. Of these patients, 272 started treatment with lurasidone and constituted the study population (**Figure 2**).

Baseline demographic characteristics, toxic habits, comorbidities, and prior treatments of the study population are shown in **Table 1**. Overall, 60.3% of patients were female, resulting in a male-to-female ratio of 0.7. The median (Q1; Q3) age was 46 years (37, 56), with the majority of patients (87.9%) between the ages of 25 and 65 years. The proportion of patients with a family history of schizophrenia was 9.2%. Up to 48.9% of patients were current smokers and 39.0% were habitual drinkers. Regarding illicit drug use, cannabis, and cocaine were used by 23.2% and 20.2% respectively, while other substances such as ecstasy, methamphetamine, and heroin were much less common. Overall, the proportion of patients with toxic substance abuse was 43.8%. The most common comorbidities at baseline were hypertension (46.7%), dyslipidemia (44.5%), diabetes (30.5%), overweight (21.3%), obesity (23.9%), and stroke (14.3%). Up to 87.1% of patients in the study population had been treated with a median (Q1; Q3) of 3 antipsychotics (2, 4) other than lurasidone prior to the inclusion.

**Table 1:** Baseline demographics, toxic habits, comorbidities and previous treatments.

Demographics	(N = 272)
Age - years	
Mean (SD)	45.9 (13.1)
Median age (Q1, Q3)	46 (37–56)
Age group – years, mean (SD)	
< 18	0 (0.0)
18 to 25	14 (5.1)
25 to 45	109 (40.1)
45 to 65	130 (47.8)
≥ 65	19 (7.0)
Female sex, n (%)	164 (60.3)
Family schizophrenia, n (%) *	25 (9.2)

Toxic habits, n (%) *	
Smoking	
Ex-smoker	25 (9.2)
Current smoker	133 (48.9)
Alcohol	
Ex-drinker	4 (1.5)
Current drinker	106 (39.0)
Cannabis	
Ex-user	2 (0.7)
Current user	63 (23.2)
Cocaine	55 (20.2)
Ecstasy	5 (1.8)
Methamphetamine	1 (0.4)
Heroin	8 (2.9)
Toxic abuse	119 (43.8)

Comorbidities, n (%) *	
High blood pressure	127 (46.7)
Dyslipidemia	121 (44.5)
Diabetes	83 (30.5)
Obesity	65 (23.9)
Overweight	58 (21.3)
Stroke	39 (14.3)
Ischemic heart disease	18 (6.6)

Previous treatments	
Any previous antipsychotic, n (%)	237 (87.1)
Number of previous antipsychotics at baseline ‡	
Mean (SD)	3.09 (1.92)
Median (Q1, Q3)	3 (2-4)
Min - Max	1 - 9

*The presence of each feature was analyzed considering the window of [Birth, Inclusion +1 month]. In case of multiple values closest to Inclusion date, the ties were broken using the ascending ordering in the table (e.g., No and Yes: Yes). ^‡^The number of antipsychotics prior to inclusion date was analyzed considering the window of [Birth, Inclusion). The variables Parenteral drugs and LSD were not reported in the table since no patient with Parenteral drugs or LSD mentions were found. The variable Toxic abuse takes value TRUE if at least one ‘Yes’ was observed in Cannabis, Cocaine, Ecstasy, Methamphetamine or Heroin or if it was detected in the free text.

Q1, Q3, lower and upper quartiles; SD, standard deviation.

### Lurasidone characteristics

**Figure 3:**
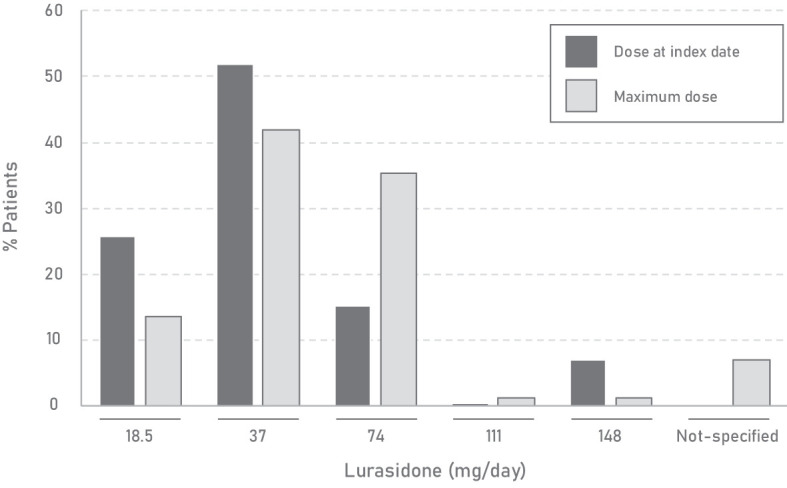
Characteristics of lurasidone doses during lurasidone treatment.

The median (Q1; Q3) time from diagnosis of a psychotic disorder to lurasidone treatment was 4.5 years (1.0; 9.7) and patients in our population were treated with the drug for a median (Q1; Q3) of 9.0 months (4.0; 14.0). The baseline and maximum doses of lurasidone achieved during the study period are shown in **Figure 3**. Before the inclusion, second-generation therapies were used much more frequently than first-generation therapies. The most common was olanzapine (52.9%), followed by quetiapine (45.2%) and aripiprazole (40.8%). Among first-generation antipsychotics, haloperidol was the most commonly prescribed (20.2%). Overall, the percentage of patients receiving antipsychotic combinations during follow-up decreased by about 31% compared with the corresponding percentage before the inclusion (87.1% and 60.3%, respectively). As a concomitant treatment, haloperidol remained the most commonly used first-generation antipsychotic (11.0%), and, among second-generation therapies, olanzapine was again the most commonly used (32.4%), followed by aripiprazole (27.2%) and quetiapine (23.5%) (**Supplementary Table 2**).

The percentage of patients with at least one relapse in the year before the inclusion was 17.6% in the monotherapy group and 16.5% in the polytherapy group. The corresponding percentages during the lurasidone treatment period were 9.3% and 26.2%, respectively.

Regarding treatment adherence, 16.9% of patients discontinued lurasidone treatment during follow-up. Almost half of the patients (43.5%) who discontinued lurasidone treatment were receiving a dose of 37 mg/day at the time of discontinuation, while 21.7% and 17.4% were receiving 74 mg/day and 18.5 mg/day, respectively, and 17.4% did not have a lurasidone dose specified when discontinuation occurred. In terms of primary diagnosis, 43.5% of patients who discontinued lurasidone had schizophrenia, 28.3% had depression, 19.6% had bipolar disorder, 4.3% had schizoaffective disorder and 4.3% had a not-specified diagnosis. Drug discontinuation rate was lower in patients treated with monotherapy than in those treated with polytherapy (41.3% vs. 58.7%). The median drug retention time for lurasidone was not reached during follow-up. The drug retention rate at 30 months was 65.5% (**Supplementary Figure 1**).

### Symptoms

**Figure 4:**
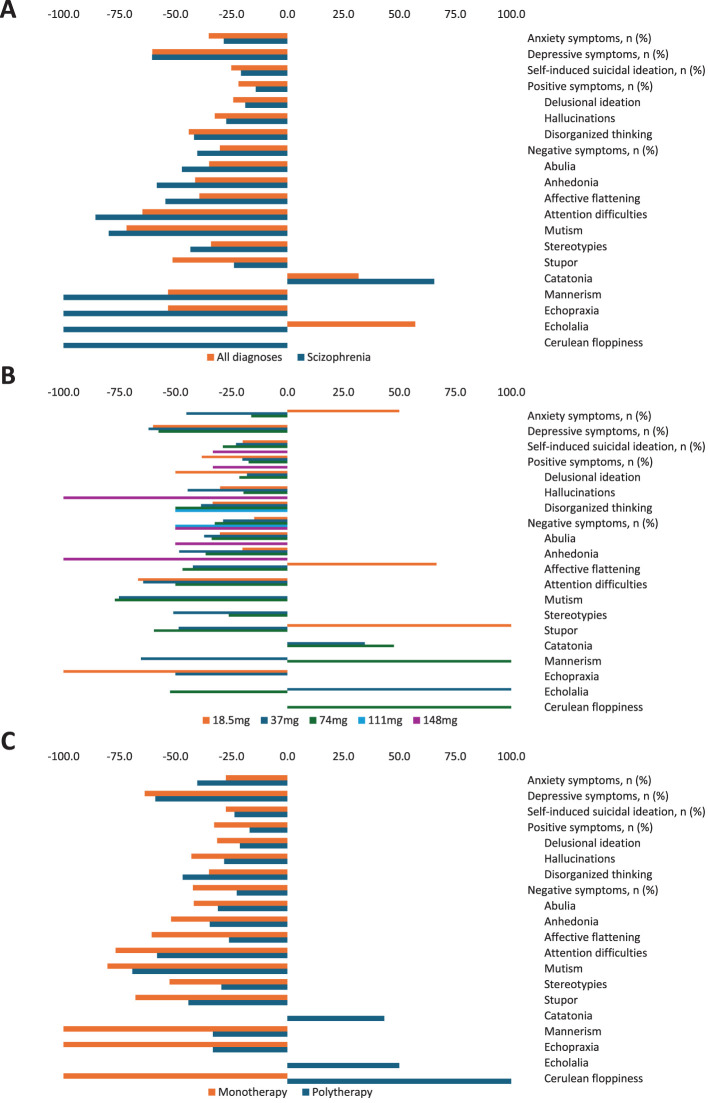
**(A)** Signs and symptoms variations between baseline and during lurasidone treatment by primary diagnosis, **(B)** maximum dose, and **(C)** type of treatment.

Lurasidone treatment markedly reduced the frequency of positive symptoms (‒21.8%) and negative symptoms (‒30.1%). However, for catatonia and echolalia, the number of patients at baseline and during follow-up was too small to assess this reduction. Lurasidone treatment also achieved important reductions in anxiety (‒35.1%), depression (‒60.3%), and suicidal ideation (‒25.0%) frequencies. When stratified by primary diagnosis, lurasidone treatment produced a comparable pattern of reductions in signs and symptoms in patients with schizophrenia (**Figure 4A**; **Supplementary Table 3**).

Although there was no apparent direct relationship between lurasidone dose and a decrease in the frequency of symptoms given the small sample size in the higher dose groups, it appeared that the greatest improvement in signs and symptoms from baseline was generally achieved with the highest dose used (**Figure 4B**; **Supplementary Table 4**). When stratified by treatment type, monotherapy was associated with a greater improvement than polytherapy for most symptoms, including positive symptoms such as delusional ideation or hallucinations, and most negative symptoms, including affective flattening, anhedonia, or abulia. Greater improvements were also achieved with monotherapy for depression and suicidal ideation. On the other hand, polytherapy was better than monotherapy in improving symptoms such as disorganized thinking and anxiety (**Figure 4C**; **Supplementary Table 5**).

### Potential adverse events

Potential treatment-emergent adverse events at baseline and during follow-up are shown in **Table 2**. Among those occurring in ≥10% of patients, the most common were paresthesia (23.5%), obesity (22.8%), and hypercholesterolemia (22.4%). Lurasidone treatment substantially reduced all adverse events, except for rhabdomyolysis, leukopenia, and neuroleptic malignant syndrome, which did not change during follow-up. Overall, lurasidone reduced up to 80% of all recorded potential adverse events by at least ‒50% (**Supplementary Table 6**). Moreover, a few comorbidities first occurred during lurasidone treatment (**Supplementary Table 7**).

**Table 2:** Potential treatment-related adverse events at baseline and during follow-up.

Adverse event, n (%)	Baseline (N = 272)	Follow-up (N = 272)	Percentage change
Paresthesia	64 (23.5)	8 (2.9)	—87.7%
Obesity	62 (22.8)	32 (11.8)	—48.2%
Hypercholesterolemia	61 (22.4)	29 (10.7)	—52.2%
Tachycardia	56 (20.6)	20 (7.4)	—64.1%
Overweight	56 (20.6)	14 (5.1)	—75.2%
Akathisia	48 (17.6)	21 (7.7)	—56.3%
Weight loss	48 (17.6)	17 (6.2)	—64.8%
Anemia	42 (15.4)	21 (7.7)	—50.0%
Hypertriglyceridemia	34 (12.5)	11 (4.0)	—68.0%
Arrhythmia	33 (12.1)	7 (2.6)	—78.5%
Hyponatremia	31 (11.4)	12 (4.4)	—61.4%
Extrapyramidal syndrome	26 (9.6)	11 (4.0)	—58.3%
Dizziness	25 (9.2)	7 (2.6)	—71.7%
Hyperglycemia	23 (8.5)	8 (2.9)	—65.9%
Deep vein thrombosis	16 (5.9)	9 (3.3)	—44.1%
Bradycardia	16 (5.9)	5 (1.8)	—69.5%
High creatine phosphokinase	15 (5.5)	>4 (1.5)	—72.7%
Pulmonary Thromboembolism	14 (5.1)	8 (2.9)	—43.1%
Erectile dysfunction	13 (4.8)	1 (0.4)	—91.7%
Galactorrhea	12 (4.4)	2 (0.7)	—84.1%
Restless legs syndrome	8 (2.9)	1 (0.4)	—86.2%
Dysmenorrhea	7 (2.6)	2 (0.7)	—73.1%
Rhabdomyolysis	5 (1.8)	5 (1.8)	0.0%
Acute kidney disease	5 (1.8)	0 (0.0)	—100.0%
Tardive dyskinesia	4 (1.5)	1 (0.4)	—73.3%
Angioedema	4 (1.5)	2 (0.7)	—53.3%
Leukopenia	4 (1.5)	4 (1.5)	0.0%
Orthostatic hypotension	3 (1.1)	1 (0.4)	—63.6%
Psychomotor hyperactivity	3 (1.1)	0 (0.0)	—100.0%
Anaphylaxis	2 (0.7)	0 (0.0)	—100.0%
Neuroleptic malignant syndrome	1 (0.4)	1 (0.4)	0.0%
Sudden death	1 (0.4)	0 (0.0)	—100.0%
Myoglobinuria	1 (0.4)	0 (0.0)	—100.0%
Stevens Johnson syndrome	1 (0.4)	0 (0.0)	—100.0%
Eosinophilia	0 (0.0)	0 (0.0)	―

The presence of each feature was analyzed considering the window of [Birth, Inclusion) and ([Inclusion + 3 months, end of follow-up]), respectively

## Discussion

In this real-world study using NLP and ML techniques, we established a large cohort of 272 real-life patients with psychiatric disorders who were treated with lurasidone across four Spanish hospitals. The median age of our cohort was 46 years and no patients younger than 18 years were detected, consistent with lurasidone unapproved status in adolescent patients at the start of the study. The most common primary diagnoses were schizophrenia, depression, and bipolar disorder, with differences in age and sex demographics aligned with recent publications (36, 37). For example, we observed a notable predominance of women, which likely reflects the higher prevalence of certain psychiatric disorders among females reported in the literature, such as major depressive disorder (38). Almost half of the patients were using substances, mainly cannabis, which is known to increase the risk of relapse, depressive symptoms, and suicidal behavior after the first episode of psychosis (39, 40). Moreover, substance use, particularly cannabis and synthetic cannabinoids, have been identified as key predictors of readmission in young patients following a psychotic episode, underscoring the need for targeted interventions in this population (41).

In terms of comorbidities, patients with schizophrenia and bipolar disorder are at a higher risk of developing metabolic syndrome and diabetes compared to the general population (42–44). However, the prevalence of cardiometabolic risk in these patients is likely underestimated due to underdiagnosis and undertreatment (45). We observed that a significant percentage of our cohort had conditions like hypertension (46.7%), dyslipidemia (44.5%), diabetes (30.5%), obesity (23.9%), and stroke (14.3%) at inclusion, which supports lurasidone’s preference among patients with such comorbidities due to its favorable metabolic profile (46). This is important because, unlike other antipsychotics such as olanzapine or quetiapine, lurasidone contributes to weight loss in patients with psychiatric disorders and comorbid obesity (47, 48). This was seen in our study with a decreased number of patients reporting obesity during lurasidone treatment.

When looking at lurasidone use, the predominant dosage was 37 mg/day, followed by 74 mg/day, with minimal utilization of 111 mg/day and 148 mg/day (<1.2%). This observation was independent of the primary diagnosis, the occurrence of psychiatric hospitalizations, or the use of lurasidone as monotherapy or polytherapy. The recommended starting dose of lurasidone in schizophrenia patients is 37 mg/day, which may be increased to a maximum of 148 mg/day based on physician judgment and observed clinical response (4). Although the starting dose of lurasidone has demonstrated significant efficacy in reducing the Positive and Negative Syndrome Scale (PANSS) total score, some studies indicate that patients with suboptimal symptom control at lower doses can benefit from higher doses (15, 49). In particular, higher doses (111-148 mg/day) have shown greater efficacy than lower doses (37-74 mg/day) in patients with acute psychotic symptoms of schizophrenia and severe agitation at baseline (50), and this high dose of lurasidone has not been associated with increased weight gain or adverse effects (51). Moreover, specific dose-related anti-hostility effects have been also described (52). This provides evidence that lurasidone is often prescribed at dosages below the recommended levels in Spain, particularly in patients with schizophrenia, which could have limited its effectiveness, especially in patients who failed other antipsychotic therapies. Although the data from this study do not allow us to fully explore the specific reasons behind the administered doses, some potential factors might include the preference for lower doses to minimize side effects, address comorbidities, or comply with institutional barriers such as formulary restrictions and cost. Additionally, lower doses might have been used as adjunctive therapy to manage residual symptoms while reducing the risk of adverse effects associated with other treatments like olanzapine (53). Furthermore, the inclusion of conditions other than schizophrenia in our study where lower doses are typically prescribed may also have contributed to this observation (54).

Long-term effectiveness of antipsychotic drugs is critical considering that more than 80% of patients with schizophrenia experience relapse within the first 5 years of treatment (55). While the benefits of long-acting injectable (LAI) antipsychotics in reducing readmission rates following a first episode of psychosis are well-documented (56, 57), there is other reference highlighting that LAIs and oral antipshychotics did not differ significantly regarding relapse prevention/hospitalization and acceptability (58). Additional evidence supports the efficacy of lurasidone in achieving higher remission rates and reducing the risk of hospitalization compared to quetiapine, which was the second most commonly used antipsychotic in our study prior to lurasidone (41, 59). Furthermore, in bipolar patients, hospitalization risk has been shown to be higher with olanzapine or aripiprazole, underscoring the potential advantages of lurasidone in this population (60). In our whole population, the relapse rate for patients on lurasidone monotherapy was notably lower after the inclusion date (17.6% vs. 9.3%), whereas it increased for those on polytherapy (16.5% vs. 26.2%). While antipsychotic monotherapy is preferred over polytherapy whenever possible [(61) and references therein] and shows better retention rates (62, 63), polytherapy has demonstrated superiority in reducing the likelihood of mortality and hospitalization risk in schizophrenia in patients with second-generation antipsychotics. In our study, patients receiving monotherapy showed lower discontinuation rates than patients receiving polytherapy (41.3% vs. 58.7%). Moreover, when stratified by treatment type, lurasidone monotherapy exhibited superior improvement over polytherapy for most symptoms, including positive and negative, as well as depression and suicidal ideation. In this context, the role of lurasidone in non-core symptoms such as depression and anhedonia has been previously described (64–66). Finally, lurasidone retention after 30 months of follow-up surpassed that reported in other studies, possibly reflecting its favorable efficacy, tolerability, and patient satisfaction, highlighting its acceptability profile (67–71). In this regard, while lurasidone has demonstrated efficacy in both monotherapy and combination therapy, we observed that monotherapy offers several distinct advantages.

On the other hand, our results showed a lack of clear dose-response relationship, possibly due to the complex pharmacodynamics of lurasidone. Variability in patient metabolism, partial adherence, and potential drug-drug interactions may also contribute to this phenomenon (72, 73). However, an independent analysis (51) demonstrated that while the efficacy of lurasidone follows a dose-response pattern in terms of efficacy, this relationship does not extend to its adverse events, underscoring the need for further research into optimized dosing and personalized treatment approaches. Additionally, individualized dosing strategies tailored to patient-specific factors such as comorbidities, treatment history, and tolerability further complicate the dose-response relationship. Lurasidone alleviated both positive and negative symptoms along with depressive and anxiety symptoms; however, we noted a slight increase in the proportion of patients with catatonia during follow-up —a common adverse effect induced by antipsychotics [(74) and references therein]. Comparative efficacy studies suggest no significant differences among available antipsychotics for positive symptoms and disorganization, except for the superior efficacy of clozapine in treating treatment-resistant schizophrenia (75). Interestingly, a retrospective chart review revealed positive outcomes for most patients with treatment-resistant schizophrenia who received a combination of lurasidone and clozapine, showing reductions in positive, depressive, and anxiety symptoms, alongside improvements in psychosocial functioning in a real-world setting (31). Therefore, the combination of lurasidone and clozapine could be an optimal polytherapy strategy for those patients with treatment-resistant schizophrenia.

Despite similar efficacy, antipsychotics exhibit diverse adverse effects. Lurasidone stands out for its favorable tolerability profile, with minimal effects on metabolic parameters and weight gain, although it carries a modest risk of extrapyramidal and other side effects (76). Notably, all major metabolic and CV adverse events were reduced during lurasidone treatment compared to the pre-treatment profile. In general practice, however, lurasidone is combined with other antipsychotics that may worsen the comorbid state of patients.

The main strength of this multicenter study is the use of innovative technology to extract and interpret large-scale RWD, providing useful information of lurasidone usage in Spain. These methods were specifically employed with rigorous validation steps and the inclusion of comprehensive patients’ clinical information across multiple hospital departments, ensuring a more reliable and multidisciplinary perspective of the patient population. This approach is different from prospective studies, where strict inclusion criteria are applied, and patient selection is more controlled. Then, our design aimed to minimize selection bias by capturing a broad, real-world cohort that reflects the diverse patient population receiving lurasidone treatment in routine clinical practice. However, limitations exist, including reliance on structured data and free-text narratives recorded in EHRs which might be constrained by the accuracy of physicians’ descriptions. This limitation could account for patient loss during follow-up and missing data points across variables. While our multicenter approach optimizes an accurate and representative characterization of patients treated with lurasidone in real-world settings, variations in the EHR systems and data collection across sites may introduce data heterogeneity. We also recognize that the inclusion of data exclusively from centers in Spain may limit the generalizability of our findings to other healthcare systems. The Spanish healthcare system, characterized by its publicly funded, universal coverage and standardized use of EHRs, may differ from systems with less uniform access to care or fragmented data infrastructures, potentially affecting the applicability of our results in other contexts. Additionally, we acknowledge that the complex clinical profiles of patients with schizophrenia make it inherently difficult to isolate the impact of any single treatment. However, the objective of this study was purely descriptive, and future research will be needed to compare different patient groups while appropriately balancing confounding factors. Finally, our methodology does not allow us to estimate causal effects.

In conclusion, by applying NLP to the free text in the EHRs of patients treated with lurasidone, we obtained a real-world picture of lurasidone use in Spain which helps fill a large research gap. Lurasidone effectively reduced the frequency of positive and negative symptoms maintaining a good safety profile, even at high doses, in monotherapy or polytherapy. This efficacy and safety spanned all pathologies studied, not just schizophrenia. Despite its positive outcomes and low discontinuation rates, lurasidone dosing in Spanish clinical practice remains conservative, indicating a need for optimized dosing strategies to enhance patient outcomes. Future clinical trials and larger prospective studies are warranted to evaluate the comparative effectiveness of lurasidone in specific subpopulations and to further validate its role in both monotherapy and polytherapy contexts, as well as studies that explore the reasons behind underdosing of the drug in real-world settings.

## Data Availability

Data can be shared on reasonable request to the corresponding author after permission has been obtained from the institutions involved.

## Group members of Savanah Research Group

Carlo Sguera, Daniel Salvador, David Casadevall, Eduard Sarró, Eva Castillo, Ignacio Salcedo, Judith Marín-Corral, Miren Taberna, Natalia Polo, Paulo Luz, and Sebastian Menke.
