# Supplementary material for: Lurasidone uses and dosages in Spain: RETROLUR, a real-world retrospective analysis using artificial intelligence
Source: Front Psychiatry. 2025 Feb 5;15:1506142. doi: 10.3389/fpsyt.2024.1506142 (PMC11862477; doi:10.3389/fpsyt.2024.1506142)
Supplement: Supplementary file 1 [file DataSheet1.docx]

Supplementary Material

**Lurasidone uses and dosages in Spain: RETROLUR, a real‑world retrospective analysis using artificial intelligence**

# Supplementary Data

## Supplementary Methods

### Study design and data source

The study was conducted in 4 hospitals of the Spanish National Health Network: Hospital Universitari Son Espases (Palma de Mallorca, Spain), Hospital Universitario Infanta Sofía (Madrid, Spain), Hospital Universitario Infanta Leonor (Madrid, Spain), and Hospital Universitario Regional de Málaga Carlos Haya (Málaga, Spain). Data from patients included in the study was unstructured free-text and structured information in EHRs, including admission and discharge notes, outpatient clinic notes, medical reports, emergency notes, procedure notes, prescriptions, and notes from all available services and departments in each participating site. The received information from different data sources was refined during different stages of the data integration process until it was integrated into a secure ecosystem. At each stage, we had intermediate information that allowed the system to determine data status, getting metrics on quality and completeness to ensure the eligibility of the participating centers. Images, such as hand-drawn pictures and scanned images, were not extracted. No data entry by physicians or their delegates into an electronic data capture platform was performed. No Clinical Research Documents were collected for this study.

### Sample size and calculation

The goal of the present study was purely descriptive, and the sample size calculation was aimed at providing the expected precision levels when estimating different feature measurements within the study population. To this end, the size of the study population was estimated considering the total number of patients covered by the participating hospitals and the number of patients receiving lurasidone in Spain during the study period. According to the report on the consumption of antipsychotic drugs published by the Spanish Ministry of Health (<https://www.aemps.gob.es/medicamentos-de-uso-humano/observatorio-de-uso-de-medicamentos/informes/>), the defined daily dose per 1000 inhabitants (DHD) of lurasidone for the years 2019 to 2021 was considered indicative of incident patients on those years, given the drug’s approval in 2019 . The DHD from 2019, 2020 and 2021 were 0.01, 0.06 and 0.1/1000 inhabitants, respectively. To estimate the size of the study population, the DHD for 2021 was used as a conservative estimate of the total number of treated patients during the study period in Spain. Our estimations were performed under the following assumptions: i) 1 daily dose equals 1 patient receiving lurasidone, ii) the number is equivalent to a cumulative incidence rate of new users during the study period, iii) the rate of treatment discontinuation is 0 (i.e. 100% of patients that started in previous years contribute to the 2021 rate), iv) the rate of lurasidone usage is homogeneous across Spain (and hence across all participating hospitals), and v) the participating hospitals represent a random sample of the Spanish population. Taking the above under consideration and assuming that each participating hospital covers a population of 350-500.000 inhabitants, we expected to observe a total of 35-50 new lurasidone treated patients per site in the whole study period, given a total of 140-200 patients. This sample size would allow us to estimate feature frequencies (with 95% confidence) with the following precision (percent units per feature frequency): 4.16-4.81 for 10%, 5.55-6.41 for 20%, 6.36-7.35 for 30%, 6.80-7.85 for 40%, and 6.93-8.01 for 50%.

### Data acquisition

The data acquisition was responsibility of the participating sites. Specific documentation listing the necessary data sources to construct the study database was created based on the study protocol.

### Data integration

Medsavana S.L. received the EHRs from heterogeneous sources, as every site could have different Information Systems. This information was then uploaded to a secure file transfer protocol utility exclusively available for each site. In the integration stage, the EHRs were included in an inventory to be prepared for the NLP phase (*EHRead*® technology). This step comprised format standardization, data cleaning, data quality reporting, and application of business rules.

### Data quality assessment

To assess the quality of the information gathered from EHRs, the total number of screened records and patients were analyzed per site, according to the main data sources (admission, consultation, or emergency notes) and hospital departments. This overview was used to determine whether records and/or patients were eligible for inclusion in terms of completeness. Sites remained anonymous and undisclosed in this section.

### Extraction of the unstructured information from electronic health records

Medsavana developed the EHRead® technology, an integrated suite of diverse Python-based modules that leverage various computational techniques, with a strong emphasis on NLP, for extracting clinically relevant information from Electronic Health Records’ (EHR) free text and providing the information in a structured database. EHRead® is capable of meaningfully interpreting the content included in the free text in various languages (Spanish, Catalan, English, French or German), regardless of the EHR system on which it operates. Then, in this study, all variables were extracted from patients EHRs using EHRead® technology. For that, conceptual definitions for all study variables were pre-specified and aligned with clinical entities found in the SNOMED Clinical Terms (a comprehensive, computationally processable collection of medical terms utilized in clinical documentation) using the SNOMED CT browser. This step facilitated the conversion of unstructured data from various hospital departments into actionable variables for extraction. EHRead®’s modules are integrated into a high-throughput big data processing pipeline, utilizing highly parallelized cloud computing with Spark. While the overall pipeline is proprietary, ML models may incorporate open-source architectures such as transformers, and models are fine-tuned for each use case to ensure optimal performance.

In this study, EHRead® was primarily applied to the dataset obtained from the participating hospitals, and the final study population was determined through the application of NLP filters. To comply with the principle of data minimization, only patients who fulfilled all inclusion and no exclusion criteria were included in the final study database which served as the foundation for subsequent analyses.

### EHRead® performance

The clinical variable extraction and processing strategy for each study variable was elaborated by a multidisciplinary task force including data scientists, medical researchers, and NLP experts. A comprehensive list of clinical variables to answer the objectives of the study was elaborated, as well as the strategies for their extraction from the various data sources during a dedicated NLP development phase. The clinical accuracy of the conceptual definitions and entity mapping was approved by medical research experts specialized in NLP who reviewed term detection summaries for false negatives and false positive detections.

To ensure the quality of data extraction, the performance of EHRead® was also externally evaluated. Specifically, this validation was carried out by external annotators following a peer-reviewed method (1). Briefly, the external annotators created the ‘standard’ to which EHRead® technology’s variable detections were compared. The aim was to measure inter-annotator agreement (IAA) to ensure guideline consistency and parameter reliability, using these annotations as a benchmark to assess EHRead® against physician annotations.

Additionally, to determine the required minimum number of annotated EHRs, we employed the Sample Calculator for Evaluation (SLiCE®), a tool designed to calculate this based on the prevalence of key variables within the EHRs. SLiCE uses a 95% confidence level, interval widths of 10% (percentage points), and targets for precision and recall, ensuring that the estimated precision and recall are accurate within ±5% (percentage points) at a 95% confidence level.

The evaluation of the system was calculated in terms of the standard metrics of Precision, Recall, and their harmonic mean F1-Score.

- *Precision* =
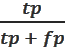
. This parameter indicates the accuracy of the system in retrieving key clinical concepts.
- *Recall* =
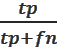
 . This parameter indicates the amount of information the system retrieves.
- *F1-Score* =
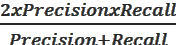
. This parameter gives us an overall performance indicator of information retrieval.

In all cases, *tp* is the number of true positives (i.e., records correctly retrieved), *fn* is the set of false negatives (i.e., records incorrectly not retrieved), and *fp* is the number of false positives (i.e., records incorrectly retrieved).

### Model development

Once the clinical entities were extracted, variables were constructed by applying dedicated data wrangling operations to their mapped entities, leveraging specific NLP parameters generated by dedicated ML models (e.g., negation, temporality, attributes, etc.) and record-specific metadata (e.g., date, medical department, record type, etc.). The NLP models used in EHRead® were iteratively optimized in a robust ML lifecycle to ensure optimal performance. General and specific models were used in this study and key processing steps included the following:

Study data capture: named-entity recognition and linking models identify clinical entities and link them with standard terminology (e.g., SNOMED CT)

Negation detection: clinical entities are classified as ‘affirmative’ and ‘non-affirmative’ (2).

Section detection: paragraphs, sentences and sub-sentences of the EHR are categorized based on clinical context (family background, patient background, or patient present).

Temporality: temporal relationships are established, linking clinical entities to time references such as absolute/explicit dates and times, as well as time differences (“last week”, “two months ago”, etc.).

Measurable parameters: Quantitative clinical data, such as test results (e.g., laboratory magnitudes) or biometric variables (e.g., body-mass index), are extracted and standardized for consistent analysis through different hospitals and services.

Specific NLP models were tailored to the study needs and included the extraction of any kind of clinical entity and/or attribute considered relevant to answer the study objectives. For its development, trained medical research experts conducted dedicated annotation projects to generate the necessary training corpora.

### Study Variables

Variable categories included sociodemographic characteristics (age, sex), toxic habits (smoking, alcohol, cannabis, cocaine, ecstasy, metamphetamine, and heroin), comorbidities, diagnosis (primary diagnosis and subtypes), lurasidone characteristics (initial and maximal doses) and other previous and concomitant first and second antipsychotic treatments (haloperidol, zuclopenthixol, chlorpromazine, fluphenazine, pipotiazine and olanzapine, quetiapine, aripiprazole, paliperidone, risperidone, clotiapine, clozapine, ziprasidone and cariprazine, respectively). Effectiveness was evaluated by estimating the percentage of change of the signs and symptoms between baseline and follow up as follows: [(% of patients with the sign or symptom at follow up - % of patients with the sign or symptom at baseline) / % of patients with the sign or symptom at baseline)] x 100). Signs and symptoms included anxiety disorder, anxious depressive syndrome, self-induced suicidal ideation, and positive and negative symptoms. Safety outcomes were evaluated by the presence of potential treatment ‑related adverse events or the first occurrence of comorbidities in different time windows (before diagnosis, before lurasidone treatment, and during lurasidone treatment).

### Statistical Analysis

The number of patients with missing data points is shown in the analysis for discrete and continuous variables. Missing data were handled according to the nature of the data collection process and based on the type of variable. In this context, following considerations were assumed:

Boolean variables: variables whose values can only be TRUE (the variable is detected in the EHR within the specified time window) or FALSE (the variable is not detected in the EHR within the specified time window). It is assumed that they are always reported if present. Therefore, missing values of this class were imputed as absences. Common examples of boolean variables include comorbidities and symptoms (e.g., at a given moment in time when a patient is visited by the physician, they can either have or not have “arterial hypertension” or “cough”).

Categorical variables: variables that can take up multiple (≥2) different values. It is assumed that these variables may not be always reflected by physicians and consequently, their absence was not imputed and was reported as missing data. One example are lifestyle habits, e.g., a patient can be a “never smoker”, a “former smoker” or a “current smoker”. Scales or scores for disease classifications or gradations where categories are not equally spaced are also included in this class (e.g., Glasgow scale, SOFA score). Unless otherwise specified, missing values for categorical variables are reported and their number is included in percentage calculations.

- Numerical variables: variables whose values are amenable for quantitative analysis (e.g., may be summarized using mean and standard deviation). These variables can be captured either in free text through specific NLP models that detect one or more terms (variable) associated with a numeric value (and its units, when applicable), or in structured data (e.g., laboratory values contained in a structured format). Unless otherwise specified, summary statistics of numerical variables are calculated based only on available values. In this study no imputation strategies for missing data were planned for numeric variables.

# Supplementary Figures and Tables

## Supplementary Figures


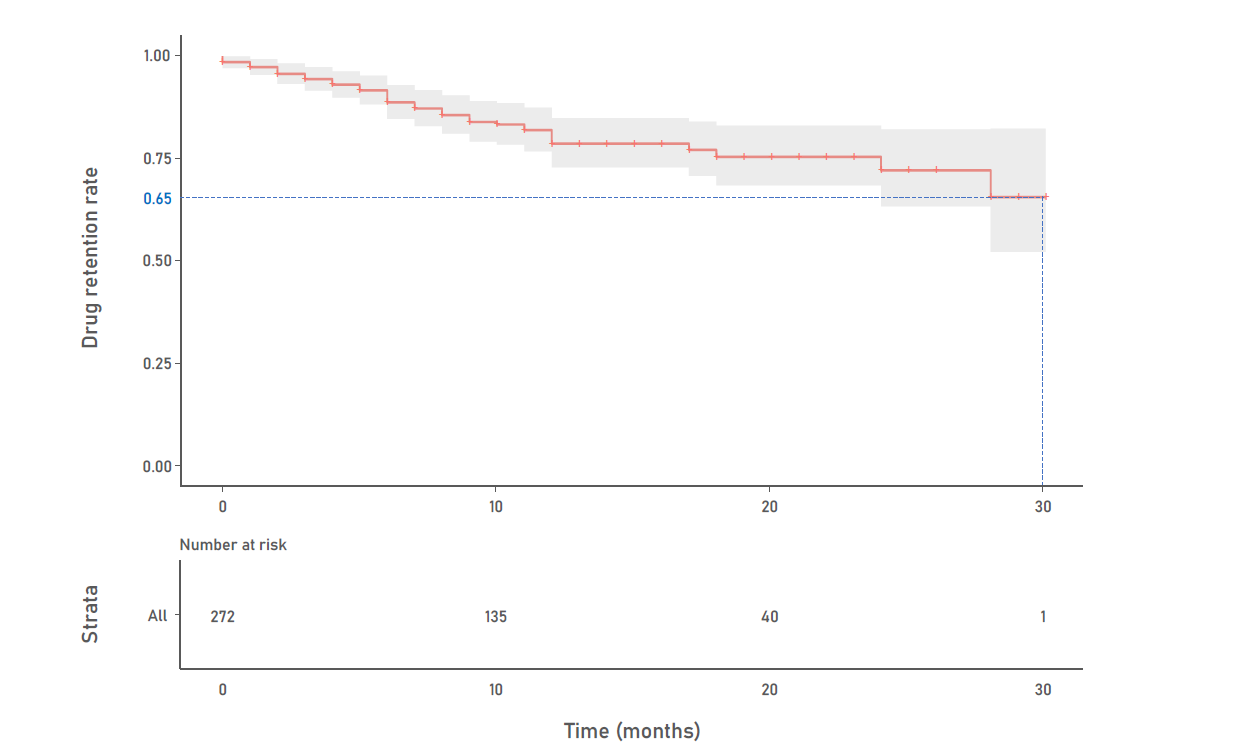


Supplementary Figure S1. Lurasidone retention rate during follow-up.

## Supplementary Tables

**Supplementary Table 1.** Performance of EHRead® identifying records that contained key variables.

| **Variable** | **Precision** | **Recall** | **F1‑score** |
| --- | --- | --- | --- |
| Schizophrenia | 0.982833 | 0.991342 | 0.987069 |
| Schizoaffective disorder | 1 | 0.946809 | 0.972678 |
| Bipolar disorder | 0.989011 | 0.947368 | 0.967742 |
| Schizophreniform disorder | 0.875 | 1 | 0.933333 |
| Psychotic attack | 0.448276 | 0.979452 | 0.615054 |
| Bipolar depression | 1 | 1 | 1 |

**Supplementary Table 2.** Previous and concomitant antipsychotics.

|  | **Previous**  **(N = 272)** | **Concomitant**  **(N = 272)** |
| --- | --- | --- |
| Any simultaneous antipsychotic, n (%) † | 237 (87.1) | 164 (60.3) |
| First-generation antipsychotics, n (%) |  |  |
| Haloperidol | 55 (20.2) | 30 (11.0) |
| Zuclopenthixol | 10 (3.7) | 6 (2.2) |
| Chlorpromazine | 5 (1.8) | 1 (0.4) |
| Fluphenazine | 1 (0.4) | 0 (0.0) |
| Pipotiazine | 0 (0.0) | 0 (0.0) |
| Second-generation antipsychotics, n (%) |  |  |
| Olanzapine | 144 (52.9) | 88 (32.4) |
| Quetiapine | 123 (45.2) | 64 (23.5) |
| Aripiprazole | 111 (40.8) | 74 (27.2) |
| Paliperidone | 91 (33.5) | 56 (20.6) |
| Risperidone | 80 (29.4) | 44 (16.2) |
| Clotiapine | 49 (18.0) | 24 (8.8) |
| Clozapine | 26 (9.6) | 23 (8.5) |
| Ziprasidone | 25 (9.2) | 10 (3.7) |
| Cariprazine | 12 (4.4) | 14 (5.1) |
| *†Antipsychotics were analyzed considering the following windows: Previous [Birth, Inclusion). Concomitant: (Inclusion + 3 months, end of lurasidone treatment). Antipsychotics detected in patients on a single date and/or at no time in a psychiatric unit were not counted.* | | |

**Supplementary Table 3.** Signs and symptoms at baseline and during follow-up by primary diagnosis.

|  | **Schizophrenia**  **(N = 95)** | | **Schizoaffective disorder**  **(N = 12)** | | **Bipolar**  **disorder**  **(N = 53)** | | **Psychotic**  **episode**  **(N = 13)** | | **Depression**  **(N = 83)** | | **Missing**  **(N = 16)** | | **Total**  **(N = 272)** | |
| --- | --- | --- | --- | --- | --- | --- | --- | --- | --- | --- | --- | --- | --- | --- |
|  | **Baseline** | **FU** | **Baseline** | **FU** | **Baseline** | **FU** | **Baseline** | **FU** | **Baseline** | **FU** | **Baseline** | **FU** | **Baseline** | **FU** |
| Anxiety symptoms, n (%) | 7 (7.4) | 5 (5.3) | 4 (33.3) | 1 (8.3) | 4 (7.5) | 3 (5.7) | 1 (7.7) | 0 (0.0) | 14 (16.9) | 10 (12.0) | 1 (6.2) | 1 (6.2) | 31 (11.4) | 20 (7.4) |
| Depressive symptoms, n (%) | 5 (5.3) | 2 (2.1) | 1 (8.3) | 0 (0.0) | 11 (20.8) | 6 (11.3) | 3 (23.1) | 2 (15.4) | 13 (15.7) | 3 (3.6) | 0 (0.0) | 0 (0.0) | 33 (12.1) | 13 (4.8) |
| Self-induced suicidal ideation, n (%) | 68 (71.6) | 54 (56.8) | 9 (75.0) | 4 (33.3) | 38 (71.7) | 28 (52.8) | 5 (38.5) | 4 (30.8) | 67 (80.7) | 48 (57.8) | 1 (6.2) | 3 (18.8) | 188 (69.1) | 141 (51.8) |
| Positive symptoms, n (%) | 85 (89.5) | 73 (76.8) | 10 (83.3) | 11 (91.7) | 40 (75.5) | 23 (43.4) | 12 (92.3) | 12 (92.3) | 44 (53.0) | 29 (34.9) | 6 (37.5) | 6 (37.5) | 197 (72.4) | 154 (56.6) |
| Delusional ideation | 80 (84.2) | 65 (68.4) | 10 (83.3) | 9 (75.0) | 36 (67.9) | 21 (39.6) | 10 (76.9) | 10 (76.9) | 36 (43.4) | 24 (28.9) | 6 (37.5) | 6 (37.5) | 178 (65.4) | 135 (49.6) |
| Hallucinations | 66 (69.5) | 48 (50.5) | 4 (33.3) | 5 (41.7) | 20 (37.7) | 9 (17.0) | 4 (30.8) | 6 (46.2) | 23 (27.7) | 11 (13.3) | 3 (18.8) | 2 (12.5) | 120 (44.1) | 81 (29.8) |
| Disorganized thinking | 36 (37.9) | 21 (22.1) | 2 (16.7) | 0 (0.0) | 13 (24.5) | 6 (11.3) | 4 (30.8) | 4 (30.8) | 11 (13.3) | 5 (6.0) | 0 (0.0) | 1 (6.2) | 66 (24.3) | 37 (13.6) |
| Negative symptoms, n (%) | 72 (75.8) | 43 (45.3) | 9 (75.0) | 7 (58.3) | 41 (77.4) | 32 (60.4) | 9 (69.2) | 10 (76.9) | 69 (83.1) | 47 (56.6) | 6 (37.5) | 5 (31.2) | 206 (75.7) | 144 (52.9) |
| Abulia | 51 (53.7) | 27 (28.4) | 5 (41.7) | 4 (33.3) | 33 (62.3) | 25 (47.2) | 2 (15.4) | 4 (30.8) | 61 (73.5) | 38 (45.8) | 3 (18.8) | 3 (18.8) | 155 (57.0) | 101 (37.1) |
| Anhedonia | 36 (37.9) | 15 (15.8) | 4 (33.3) | 2 (16.7) | 30 (56.6) | 22 (41.5) | 4 (30.8) | 3 (23.1) | 51 (61.4) | 28 (33.7) | 1 (6.2) | 4 (25.0) | 126 (46.3) | 74 (27.2) |
| Affective flattening | 33 (34.7) | 15 (15.8) | 7 (58.3) | 5 (41.7) | 14 (26.4) | 8 (15.1) | 1 (7.7) | 1 (7.7) | 17 (20.5) | 13 (15.7) | 2 (12.5) | 3 (18.8) | 74 (27.2) | 45 (16.5) |
| Attention difficulties | 14 (14.7) | 2 (2.1) | 0 (0.0) | 0 (0.0) | 8 (15.1) | 2 (3.8) | 2 (15.4) | 2 (15.4) | 12 (14.5) | 7 (8.4) | 1 (6.2) | 0 (0.0) | 37 (13.6) | 13 (4.8) |
| Mutism | 15 (15.8) | 3 (3.2) | 0 (0.0) | 1 (8.3) | 6 (11.3) | 1 (1.9) | 2 (15.4) | 1 (7.7) | 4 (4.8) | 2 (2.4) | 1 (6.2) | 0 (0.0) | 28 (10.3) | 8 (2.9) |
| Stereotypies | 7 (7.4) | 4 (4.2) | 0 (0.0) | 0 (0.0) | 0 (0.0) | 0 (0.0) | 1 (7.7) | 2 (15.4) | 4 (4.8) | 1 (1.2) | 0 (0.0) | 1 (6.2) | 12 (4.4) | 8 (2.9) |
| Stupor | 4 (4.2) | 3 (3.2) | 0 (0.0) | 0 (0.0) | 3 (5.7) | 0 (0.0) | 1 (7.7) | 1 (7.7) | 2 (2.4) | 1 (1.2) | 0 (0.0) | 0 (0.0) | 10 (3.7) | 5 (1.8) |
| Catatonia | 3 (3.2) | 5 (5.3) | 0 (0.0) | 0 (0.0) | 2 (3.8) | 1 (1.9) | 0 (0.0) | 0 (0.0) | 1 (1.2) | 2 (2.4) | 0 (0.0) | 0 (0.0) | 6 (2.2) | 8 (2.9) |
| Mannerism | 3 (3.2) | 0 (0.0) | 1 (8.3) | 0 (0.0) | 0 (0.0) | 0 (0.0) | 0 (0.0) | 1 (7.7) | 0 (0.0) | 0 (0.0) | 0 (0.0) | 1 (6.2) | 4 (1.5) | 2 (0.7) |
| Echopraxia | 2 (2.1) | 0 (0.0) | 0 (0.0) | 0 (0.0) | 1 (1.9) | 0 (0.0) | 1 (7.7) | 1 (7.7) | 0 (0.0) | 1 (1.2) | 0 (0.0) | 0 (0.0) | 4 (1.5) | 2 (0.7) |
| Echolalia | 1 (1.1) | 0 (0.0) | 0 (0.0) | 0 (0.0) | 0 (0.0) | 0 (0.0) | 1 (7.7) | 1 (7.7) | 0 (0.0) | 2 (2.4) | 0 (0.0) | 0 (0.0) | 2 (0.7) | 3 (1.1) |
| Cerulean floppiness | 1 (1.1) | 0 (0.0) | 0 (0.0) | 0 (0.0) | 1 (1.9) | 1 (1.9) | 0 (0.0) | 1 (7.7) | 0 (0.0) | 0 (0.0) | 0 (0.0) | 0 (0.0) | 2 (0.7) | 2 (0.7) |
| *The presence of each feature was analyzed considering the window of [Birth, Inclusion + 1 month] for baseline and [Inclusion, end of follow-up) for follow-up. The negative symptoms catalepsy, catatonic negativism, and alogia are not represented in the table because they had 0 occurrences in all the groups. Abbreviations: FU, Follow-up* | | | | | | | | | | | | | | |

**Supplementary Table 4.** Psychiatric signs and symptoms at baseline and follow-up stratified by maximum dose.

|  | **18.5 mg**  **(N = 37)** | | **37 mg**  **(N = 114)** | | **74 mg**  **(N = 96)** | | **111 mg**  **(N = 3)** | | **148 mg**  **(N = 3)** | | **Missing**  **(N = 19)** | | **Total**  **(N = 272)** | |
| --- | --- | --- | --- | --- | --- | --- | --- | --- | --- | --- | --- | --- | --- | --- |
|  | **Baseline** | **FU** | **Baseline** | **FU** | **Baseline** | **FU** | **Baseline** | **FU** | **Baseline** | **FU** | **Baseline** | **FU** | **Baseline** | **FU** |
| Anxiety symptoms, n (%) | 2 (5.4) | 3 (8.1) | 20 (17.5) | 11 (9.6) | 6 (6.2) | 5 (5.2) | 0 (0.0) | 0 (0.0) | 0 (0.0) | 0 (0.0) | 3 (15.8) | 1 (5.3) | 31 (11.4) | 20 (7.4) |
| Depressive symptoms, n (%) | 5 (13.5) | 2 (5.4) | 21 (18.4) | 8 (7.0) | 7 (7.3) | 3 (3.1) | 0 (0.0) | 0 (0.0) | 0 (0.0) | 0 (0.0) | 0 (0.0) | 0 (0.0) | 33 (12.1) | 13 (4.8) |
| Self-induced suicidal ideation, n (%) | 25 (67.6) | 20 (54.1) | 83 (72.8) | 64 (56.1) | 66 (68.8) | 47 (49.0) | 2 (66.7) | 2 (66.7) | 3 (100.0) | 2 (66.7) | 9 (47.4) | 6 (31.6) | 188 (69.1) | 141 (51.8) |
| Positive symptoms, n (%) | 21 (56.8) | 13 (35.1) | 80 (70.2) | 64 (56.1) | 75 (78.1) | 62 (64.6) | 3 (100.0) | 3 (100.0) | 3 (100.0) | 2 (66.7) | 15 (78.9) | 10 (52.6) | 197 (72.4) | 154 (56.6) |
| Delusional ideation | 18 (48.6) | 9 (24.3) | 72 (63.2) | 59 (51.8) | 70 (72.9) | 55 (57.3) | 2 (66.7) | 2 (66.7) | 2 (66.7) | 2 (66.7) | 14 (73.7) | 8 (42.1) | 178 (65.4) | 135 (49.6) |
| Hallucinations | 10 (27.0) | 7 (18.9) | 45 (39.5) | 25 (21.9) | 51 (53.1) | 41 (42.7) | 2 (66.7) | 2 (66.7) | 3 (100.0) | 0 (0.0) | 9 (47.4) | 6 (31.6) | 120 (44.1) | 81 (29.8) |
| Disorganized thinking | 6 (16.2) | 4 (10.8) | 26 (22.8) | 16 (14.0) | 28 (29.2) | 14 (14.6) | 2 (66.7) | 1 (33.3) | 0 (0.0) | 0 (0.0) | 4 (21.1) | 2 (10.5) | 66 (24.3) | 37 (13.6) |
| Negative symptoms, n (%) | 27 (73.0) | 23 (62.2) | 87 (76.3) | 62 (54.4) | 71 (74.0) | 48 (50.0) | 2 (66.7) | 1 (33.3) | 2 (66.7) | 1 (33.3) | 17 (89.5) | 9 (47.4) | 206 (75.7) | 144 (52.9) |
| Abulia | 20 (54.1) | 14 (37.8) | 70 (61.4) | 44 (38.6) | 53 (55.2) | 35 (36.5) | 0 (0.0) | 0 (0.0) | 2 (66.7) | 1 (33.3) | 10 (52.6) | 7 (36.8) | 155 (57.0) | 101 (37.1) |
| Anhedonia | 15 (40.5) | 12 (32.4) | 62 (54.4) | 32 (28.1) | 41 (42.7) | 26 (27.1) | 0 (0.0) | 0 (0.0) | 1 (33.3) | 0 (0.0) | 7 (36.8) | 4 (21.1) | 126 (46.3) | 74 (27.2) |
| Affective flattening | 3 (8.1) | 5 (13.5) | 33 (28.9) | 19 (16.7) | 32 (33.3) | 17 (17.7) | 0 (0.0) | 0 (0.0) | 0 (0.0) | 0 (0.0) | 6 (31.6) | 4 (21.1) | 74 (27.2) | 45 (16.5) |
| Attention difficulties | 6 (16.2) | 2 (5.4) | 17 (14.9) | 6 (5.3) | 10 (10.4) | 5 (5.2) | 0 (0.0) | 0 (0.0) | 0 (0.0) | 0 (0.0) | 4 (21.1) | 0 (0.0) | 37 (13.6) | 13 (4.8) |
| Mutism | 2 (5.4) | 2 (5.4) | 12 (10.5) | 3 (2.6) | 13 (13.5) | 3 (3.1) | 0 (0.0) | 0 (0.0) | 0 (0.0) | 0 (0.0) | 1 (5.3) | 0 (0.0) | 28 (10.3) | 8 (2.9) |
| Stereotypies | 1 (2.7) | 1 (2.7) | 6 (5.3) | 3 (2.6) | 4 (4.2) | 3 (3.1) | 0 (0.0) | 0 (0.0) | 0 (0.0) | 0 (0.0) | 1 (5.3) | 1 (5.3) | 12 (4.4) | 8 (2.9) |
| Stupor | 0 (0.0) | 1 (2.7) | 4 (3.5) | 2 (1.8) | 5 (5.2) | 2 (2.1) | 0 (0.0) | 0 (0.0) | 0 (0.0) | 0 (0.0) | 1 (5.3) | 0 (0.0) | 10 (3.7) | 5 (1.8) |
| Catatonia | 0 (0.0) | 0 (0.0) | 3 (2.6) | 4 (3.5) | 2 (2.1) | 3 (3.1) | 0 (0.0) | 0 (0.0) | 0 (0.0) | 0 (0.0) | 1 (5.3) | 1 (5.3) | 6 (2.2) | 8 (2.9) |
| Mannerism | 0 (0.0) | 0 (0.0) | 3 (2.6) | 1 (0.9) | 0 (0.0) | 1 (1.0) | 0 (0.0) | 0 (0.0) | 0 (0.0) | 0 (0.0) | 1 (5.3) | 0 (0.0) | 4 (1.5) | 2 (0.7) |
| Echopraxia | 1 (2.7) | 0 (0.0) | 2 (1.8) | 1 (0.9) | 1 (1.0) | 1 (1.0) | 0 (0.0) | 0 (0.0) | 0 (0.0) | 0 (0.0) | 0 (0.0) | 0 (0.0) | 4 (1.5) | 2 (0.7) |
| Echolalia | 0 (0.0) | 0 (0.0) | 0 (0.0) | 2 (1.8) | 2 (2.1) | 1 (1.0) | 0 (0.0) | 0 (0.0) | 0 (0.0) | 0 (0.0) | 0 (0.0) | 0 (0.0) | 2 (0.7) | 3 (1.1) |
| Cerulean floppiness | 0 (0.0) | 0 (0.0) | 1 (0.9) | 1 (0.9) | 0 (0.0) | 1 (1.0) | 0 (0.0) | 0 (0.0) | 0 (0.0) | 0 (0.0) | 1 (5.3) | 0 (0.0) | 2 (0.7) | 2 (0.7) |
| *The presence of each feature was analyzed considering the window of [Birth, Inclusion + 1 month] for baseline and [Inclusion, end of follow-up) for follow-up. The negative symptoms catalepsy, catatonic negativism, and alogia are not represented in the table because they had 0 occurrences in all the groups. Abbreviations: FU, Follow-up* | | | | | | | | | | | | | | |

**Supplementary Table 5.** Psychiatric signs and symptoms at baseline and follow-up stratified by type of treatment.

|  | **Baseline** | | **Follow-up** | |
| --- | --- | --- | --- | --- |
|  | **Monotherapy**  **(N = 108)** | **Polytherapy**  **(N = 164)** | **Monotherapy**  **(N = 108)** | **Polytherapy**  **(N = 164)** |
| Anxiety symptoms, n (%) | 11 (10.2) | 20 (12.2) | 8 (7.4) | 12 (7.3) |
| Depressive symptoms, n (%) | 11 (10.2) | 22 (13.4) | 4 (3.7) | 9 (5.5) |
| Self-induced suicidal ideation, n (%) | 69 (63.9) | 119 (72.6) | 50 (46.3) | 91 (55.5) |
| Positive symptoms, n (%) | 61 (56.5) | 136 (82.9) | 41 (38.0) | 113 (68.9) |
| Delusional ideation | 51 (47.2) | 127 (77.4) | 35 (32.4) | 100 (61.0) |
| Hallucinations | 35 (32.4) | 85 (51.8) | 20 (18.5) | 61 (37.2) |
| Disorganized thinking | 17 (15.7) | 49 (29.9) | 11 (10.2) | 26 (15.9) |
| Negative symptoms, n (%) | 78 (72.2) | 128 (78.0) | 45 (41.7) | 99 (60.4) |
| Abulia | 55 (50.9) | 100 (61.0) | 32 (29.6) | 69 (42.1) |
| Anhedonia | 48 (44.4) | 78 (47.6) | 23 (21.3) | 51 (31.1) |
| Affective flattening | 28 (25.9) | 46 (28.0) | 11 (10.2) | 34 (20.7) |
| Attention difficulties | 13 (12.0) | 24 (14.6) | 3 (2.8) | 10 (6.1) |
| Mutism | 5 (4.6) | 23 (14.0) | 1 (0.9) | 7 (4.3) |
| Stereotypies | 2 (1.9) | 10 (6.1) | 1 (0.9) | 7 (4.3) |
| Stupor | 3 (2.8) | 7 (4.3) | 1 (0.9) | 4 (2.4) |
| Catatonia | 1 (0.9) | 5 (3.0) | 1 (0.9) | 7 (4.3) |
| Mannerism | 1 (0.9) | 3 (1.8) | 0 (0.0) | 2 (1.2) |
| Echopraxia | 1 (0.9) | 3 (1.8) | 0 (0.0) | 2 (1.2) |
| Echolalia | 0 (0.0) | 2 (1.2) | 0 (0.0) | 3 (1.8) |
| Cerulean floppiness | 1 (0.9) | 1 (0.6) | 0 (0.0) | 2 (1.2) |
| *The presence of each feature was analyzed considering the window of [Birth, Inclusion + 1 month] for baseline and [Inclusion, end of follow-up) for follow-up. The negative symptoms catalepsy, catatonic negativism, and alogia are not represented in the table because they had 0 occurrences in all the groups.* | | | | |

**Supplementary Table 6.** Potential treatment‑related adverse events at baseline and during follow-up.

| **Adverse event, n (%)** | **Baseline (N = 272)** | **Follow-up (N = 272)** | **Percentage change** |
| --- | --- | --- | --- |
| Paresthesia | 64 (23.5) | 8 (2.9) | —87.7% |
| Obesity | 62 (22.8) | 32 (11.8) | —48.2% |
| Hypercholesterolemia | 61 (22.4) | 29 (10.7) | —52.2% |
| Tachycardia | 56 (20.6) | 20 (7.4) | —64.1% |
| Overweight | 56 (20.6) | 14 (5.1) | —75.2% |
| Akathisia | 48 (17.6) | 21 (7.7) | —56.3% |
| Weight loss | 48 (17.6) | 17 (6.2) | —64.8% |
| Anemia | 42 (15.4) | 21 (7.7) | —50.0% |
| Hypertriglyceridemia | 34 (12.5) | 11 (4.0) | —68.0% |
| Arrhythmia | 33 (12.1) | 7 (2.6) | —78.5% |
| Hyponatremia | 31 (11.4) | 12 (4.4) | —61.4% |
| Extrapyramidal syndrome | 26 (9.6) | 11 (4.0) | —58.3% |
| Dizziness | 25 (9.2) | 7 (2.6) | —71.7% |
| Hyperglycemia | 23 (8.5) | 8 (2.9) | —65.9% |
| Deep vein thrombosis | 16 (5.9) | 9 (3.3) | —44.1% |
| Bradycardia | 16 (5.9) | 5 (1.8) | —69.5% |
| High creatine phosphokinase | 15 (5.5) | 4 (1.5) | —72.7% |
| Pulmonary Thromboembolism | 14 (5.1) | 8 (2.9) | —43.1% |
| Erectile dysfunction | 13 (4.8) | 1 (0.4) | —91.7% |
| Galactorrhea | 12 (4.4) | 2 (0.7) | —84.1% |
| Restless legs syndrome | 8 (2.9) | 1 (0.4) | —86.2% |
| Dysmenorrhea | 7 (2.6) | 2 (0.7) | —73.1% |
| Rhabdomyolysis | 5 (1.8) | 5 (1.8) | 0.0% |
| Acute kidney disease | 5 (1.8) | 0 (0.0) | —100.0% |
| Tardive dyskinesia | 4 (1.5) | 1 (0.4) | —73.3% |
| Angioedema | 4 (1.5) | 2 (0.7) | —53.3% |
| Leukopenia | 4 (1.5) | 4 (1.5) | 0.0% |
| Orthostatic hypotension | 3 (1.1) | 1 (0.4) | —63.6% |
| Psychomotor hyperactivity | 3 (1.1) | 0 (0.0) | —100.0% |
| Anaphylaxis | 2 (0.7) | 0 (0.0) | —100.0% |
| Neuroleptic malignant syndrome | 1 (0.4) | 1 (0.4) | 0.0% |
| Sudden death | 1 (0.4) | 0 (0.0) | —100.0% |
| Myoglobinuria | 1 (0.4) | 0 (0.0) | —100.0% |
| Stevens Johnson syndrome | 1 (0.4) | 0 (0.0) | —100.0% |
| Eosinophilia | 0 (0.0) | 0 (0.0) | ― |
| *The presence of each feature was analysed considering the window of [Birth, Inclusion) and ([Inclusion + 3 months, end of follow-up]), respectively.* | | | |

**Supplementary Table 7.** Comorbidities that first occurred before primary diagnosis, before lurasidone and during lurasidone treatment.

|  | **Before**  **primary diagnosis** | **Before**  **lurasidone** | **During**  **lurasidone** |
| --- | --- | --- | --- |
| High blood pressure | 48 (21.15) | 71 (31.28) | 15 (6.61) |
| Diabetes | 30 (13.22) | 46 (20.26) | 16 (7.05) |
| Dyslipidemia | 34 (14.98) | 70 (30.84) | 26 (11.45) |
| Ischemic heart disease | 5 (2.20) | 12 (5.29) | 0 (0.00) |
| Stroke | 6 (2.64) | 31 (13.66) | 6 (2.64) |
| Obesity | 18 (7.93) | 42 (18.50) | 7 (3.08) |
| Overweight | 15 (6.61) | 38 (16.74) | 10 (4.41) |
| *The presence of each feature was analysed considering the following windows: Before primary diagnosis [Birth, Diagnosis); Before lurasidone [Diagnosis, Inclusion); During lurasidone: lurasidone treatment period ([Inclusion, end lurasidone treatment]).* | | | |

# References

1. Canales L, Menke S, Marchesseau S, D'Agostino A, Del Rio-Bermudez C, Taberna M, et al. Assessing the Performance of Clinical Natural Language Processing Systems: Development of an Evaluation Methodology. JMIR Med Inform. 2021;9(7):e20492.

2. Arguello-Gonzalez G, Aquino-Esperanza J, Salvador D, Breton-Romero R, Del Rio-Bermudez C, Tello J, et al. Negation recognition in clinical natural language processing using a combination of the NegEx algorithm and a convolutional neural network. BMC Med Inform Decis Mak. 2023;23(1):216.
